# Editorial: Filamentous Bacteriophage in Bio/Nano/Technology, Bacterial Pathogenesis and Ecology

**DOI:** 10.3389/fmicb.2016.02109

**Published:** 2016-12-23

**Authors:** Jasna Rakonjac, Bhabatosh Das, Ratmir Derda

**Affiliations:** ^1^Institute of Fundamental Sciences, Massey UniversityPalmerston North, New Zealand; ^2^Maurice Wilkins Centre for Molecular Biodiscovery, The University of AucklandAuckland, New Zealand; ^3^Molecular Genetics Laboratory, Centre for Human Microbial Ecology, Translational Health Science and Technology Institute, NCR Biotech Science ClusterFaridabad, India; ^4^Department of Chemistry and Alberta Glycomics Centre, University of AlbertaEdmonton, Canada

**Keywords:** filamentous bacteriophage, phage display, bionanotechnology, phage therapy, bacterial pathogenesis, biological nanorods, microbial communities, biofilms

## Introduction

Filamentous bacteriophage predominantly infect Gram-negative bacteria and make an important contribution to host physiology, ecology, and virulence, including production of deadly toxins, such as cholera toxin. The unique filamentous structure, small genome size (4–12 kbp), replicative and/or integrative mode of inheritance, simple cultivation, and easy genomic manipulation sparked considerable attention to this class of bacteriophage for a number of applications, including cloning, sequencing, recombinant protein expression, phage display technology, and nanotechnology. This book covers a range of topics that can be grouped into two themes: impact of diverse filamentous phage on their host bacteria (five chapters) and applications of *Escherichia coli* Ff phage (eight chapters).

## Impact of diverse filamentous phage on the host bacteria

Five articles explore diverse filamentous bacteriophage, including identification, replication, integration into the host chromosome, and effect on their bacterial host properties, such as growth rate, biofilm dynamics, and virulence.

Nagayoshi et al. describe the first fully sequenced hyperthermophilic filamentous phage, ΦOH3, discovered in geothermal water. This phage infects the thermophilic bacterium *Thermus thermophilus* HB8. Ahmad et al. identify and describe a novel filamentous phage isolated from soil. The phage, named XacF1, causes loss of virulence in *Xanthomonas axonopodis pv*. citri, the causative agent of citrus canker, and could potentially be used for treatment or prevention of this disease.

Both XacF1 and ΦOH3 replicate efficiently and form turbid plaques due to increase of the host generation time, but do not kill the host. Lack of the host killing is intrinsic to the secretion-like process of filamentous phage assembly and release, while the superinfection is prevented due to the blocking of primary and secondary host receptors by the production of phage-encoded receptor-binding protein pIII in the infected cells. One exception to these universally accepted rules is prophage Pf4 of *Pseudomonas aeruginosa* PAO1. This phage converts into a “superinfective” form within the mature *P. aeruginosa* biofilms, infecting and killing the surrounding prophage-containing cells. Here, Hui et al. identify a role of reactive oxygen or nitrogen species DNA-damaging activities in the formation of superinfective phage, providing a link to the observed high-frequency mutations in the gene encoding repressor of Pf4 phage replication in the mature *P. aeruginosa* biofilms.

In contrast to the phage described above, filamentous phage YpfΦ of the plague bacillus *Yersinia pestis* replicates poorly, yet allows better colonization of the mammalian host in comparison to the phage-free strain. Derbise and Carniel review the intertwined microevolution of *Y. pestis* and YpfΦ over the past 3000 years. Some peculiarities of this phage include its broad host spectrum, elusive host receptor(s), and hard-to-reconcile pattern of seemingly exclusively episomal or integrated states in closely related *Y. pestis* strains.

Most lysogenic filamentous phage rely on a host-encoded XerCD recombinase for integration into highly conserved *dif* sites of bacterial chromosomes; however the mechanisms of integration and prophage biology vary widely. Das reviews the integration mechanisms of three lysogenic filamentous vibriophage (CTXΦ, VGJΦ, and TLCΦ) into the *Vibrio cholerae* chromosomes. Variation in DNA sequences of *attP* sites in the phage genomes drives differences in the integration and excision mechanisms, which ultimately impact on the lysogen activation, prophage replication, and efficiency of phage production. This review therefore outlines how the *attP* sites in a filamentous phage can be used to predict integration/replication modes of filamentous prophage and conversely, how the engineered *attP* sites can be used to design novel types of chromosomally-integrated bacterial expression vectors.

## Applications of the Ff filamentous phage

Eight chapters in this book review or report recent applications and technological innovations involving Ff phage of *E. coli*, or derived particles. Phage display is the most prominent application of filamentous phage. It was developed on the shoulders of versatile cloning vectors derived from the *E. coli* Ff (F-pilus specific) filamentous phage (f1, fd, and M13), and knowledge about their life cycle. Combinatorial technologies including Ff phage display are based on a physical link of coding sequence to encoded protein displayed on the virus particle. Screening vast Ff display libraries for variants that bind a “bait” of interest has resulted over the past 25 years in identification of bioactive peptides or therapeutic recombinant antibodies. Two chapters, by Gagic et al. and Henry et al., review, respectively, phage display applications for discovery of microbial surface proteins (including vaccine targets) and non-traditional applications of phage particles as therapeutic biologics, vaccines carriers, or bioconjugation scaffolds.

A technology report (Tjhung et al.) addresses an issue that has plagued phage display libraries of proteins and peptides fused to the N-terminus of virion protein pIII, in that some peptide variants are more likely to be degraded than others. Recombinant phage encoding these degradable variants have advantage at amplification step over other library clones, due to more efficient pIII-mediated infection of the host, and may outcompete the true binders in the library screens. The authors demonstrate that this can be prevented by displaying peptides between the pIII N1 and N2 domains instead of display at the N-terminus. Given that N1 domain is essential for infection, amplification of recombinant phage clones depends on preservation of displayed peptide (and thereby retention of the N1 domain in the phage). This strategy eliminates those recombinant clones in the library whose displayed peptides are degraded. It is very likely that phage display between N1 and N2 domains of pIII will be taken up by many researchers in the future.

Two research reports describe novel applications of Ff-phage-derived particles in tumor targeting. Gillespie et al. describe a new approach for assembly of tumor-targeting drug-loaded liposomes, by enabling spontaneous insertion of cancer-cell-binding peptide-pVIII fusion protein. The insertion via pVIII hydrophobic core without damaging the liposome was achieved by applying a novel method for direct purification from the phage particles, using 2-propanol. This protocol greatly simplifies the assembly of cancer-targeting drug-loaded liposomes, allowing screening of multiple peptides for targeting efficiency and drug delivery. Dor-On and Solomon report brain tumor targeting by naked Ff phage (not displaying any brain-targeting peptides) in a mouse model of glioblastoma after intranasal application. Interestingly, particle-associated lipopolysaccharides may be the key to brain targeting and anti-tumor activity of Ff in this model.

Three reports describe applications of Ff phage as nanoparticles. Sattar et al. report development of a method to functionalize and efficiently produce extremely short Ff-derived particles (50 nm in length) that contain no genes or antibiotic markers. The authors show that the short particles perform better than the full-length phage of the same composition as diagnostic particles in lateral-flow diagnostic assays. In a short review, Bernard and Francis discuss modifications that are essential for applications of Ff phage as functionalized nanoparticles. These include chemical conjugation to organic molecules such as fluorophores, pigments, carbohydrates, or inorganic molecules. One fascinating property of filamentous phage is that they are liquid crystals at high concentrations. Review by Dogic gives a clear, biologist-friendly, and up-to-date account of the liquid crystalline properties filamentous phage and their applications in the soft matter physics.

## Perspective

Future holds discovery of many novel filamentous phage. Some of these will likely be used as genetic tools for bacterial engineering, utilizing knowledge about their *attP* sites, integration, and replication. Many filamentous phage modulate bacterial pathogenicity, hence therapeutic interventions against pathogenic bacteria, based on known and novel filamentous bacteriophage, are eagerly anticipated. Filamentous phage of innocuous bacteria other than currently used Ff (f1, fd, and M13) will find applications in biotechnology, biomedicine, and nanotechnology, allowing exploration of novel properties, with the aim of decreasing the production cost and environmental footprint. Upscaling and eliminating safety concerns (removal of antibiotic-resistance genes and ability to replicate) will allow transition of filamentous-phage-particle-based technology from the laboratory containment to the consumer. In parallel, filamentous-phage-derived particles of ever more imaginative functions or physical properties will be designed and assembled into advanced nanostructures and nanomachines.

## Author contributions

Manuscript was written by JR and BD; it was edited by all three authors.

## Funding

Funding to JR laboratory by Palmerston North Medical Research Foundation, Massey University, Institute of Fundamental Sciences, Anonymous Donor and the Maurice Wilkins Centre for Molecular Biodiscovery is gratefully acknowledged. Work in BD laboratory was funded by Department of Biotechnology, Govt. of India (Grant No. BT/MB/THSTI/HMC-SFC/2011). RD acknowledges funding from the Alberta Glycomics Centre.

### Conflict of interest statement

The authors declare that the research was conducted in the absence of any commercial or financial relationships that could be construed as a potential conflict of interest.

